# Partial substitution of fish oil for linseed oil enhances beneficial fatty acids from rumen biohydrogenation but reduces ruminal fermentation and digestibility in growing goats

**DOI:** 10.1093/tas/txab116

**Published:** 2021-07-07

**Authors:** Lam Phuoc Thanh, Noppharat Phakachoed, Wisitiporn Suksombat, Juan J Loor, Tran Thi Thuy Hang

**Affiliations:** 1 Department of Animal Sciences, Can Tho University, Ninh Kieu, Can Tho 94000, Viet Nam; 2 Department of Animal Production Technology, Kalasin University, Mueang, Kalasin 46000, Thailand; 3 Technopolis, Suranaree University of Technology, Muang, Nakhon Ratchasima 30000, Thailand; 4 Department of Animal Sciences, University of Illinoi at Urbana Champaign, Urbana, IL 61801, USA; 5 Department of Agricultural Technology, Can Tho University, Phung Hiep, Hau Giang 95000, Viet Nam

**Keywords:** fatty acid biohydrogenation, fish oil, growing goat, linseed oil, ruminal fermentation

## Abstract

This study was performed to investigate effects of partial replacement of fish oil (FO) for linseed oil (LO) on digestibility, ruminal fermentation and biohydrogenation in growing goats. Experiment 1 was carried out in four growing male goats aged 6 months in a 4 × 4 Latin square design. Goats were fed a basal diet supplemented with 25 g/kg dry matter either LO alone or in combination with tuna FO. Treatments were developed by replacing FO for LO at ratios of 0, 5, 10 and 15 g/kg DM corresponding to FO-0, FO-5, FO-10 and FO-15, respectively. Experiment 2 was carried out in an *in vitro* incubation system including 12 fermenters with the same four treatments. Each fermenter consisted of 40 mL goat ruminal fluid, 160 mL warm buffer, 2 g mixed substrates, and 50 mg FO-0, FO-5, FO-10 or FO-15. Fish oil inclusion reduced (*P *< 0.05) digestibility and nitrogen retention in Experiment 1. Increasing doses of FO in the diet induced a strong drop (*P *< 0.001) in ruminal total volatile fatty acid (VFA) concentration and protozoa population at 3 h post incubation, but did not affect individual VFA proportions. Substitution of FO for LO decreased mean concentrations of C18:0 (*P *= 0.057), *c*-9,*c*-12 C18:2 and C18:3n-3 (*P *< 0.001), but increased (*P* < 0.001) C20:5n-3 and C22:6n-3. Feeding FO-10 enhanced formation of ruminal *c*-9,*t*-11 conjugated linoleic acid (CLA) concentration compared with FO-0. Overall, combined data suggest that to improve ruminal concentrations of C20:5n-3, C22:6n-3, and *c*-9,*t*-11 CLA for deposition in tissues or milk with minimal risk of affecting digestibility and ruminal fermentation, a dietary supplementation of 15 g/kg LO and 10 g/kg FO would be suitable.

## INTRODUCTION

Conjugated linoleic acids (CLA) are known to have anti-carcinogenic, anti-obesity, antioxidant and anti-inflammatory effects (Kim et al., 2016). Ruminant animal products are the richest sources of CLA (Alfaia et al., 2017), a fact that has led to vast amount of research in terms of feed formulations to enhance the production of these fatty acids (Siurana and Calsamiglia, 2016; Cabiddu et al., 2017). Biohydrogenation (BH) of linoleic acid (LA) and alpha-linolenic acid (ALA) takes place naturally in the rumen to form CLA, followed by formation of vaccenic acid (*t*-11 C18:1, VA) and then stearic acid (SA) as the final product (Bauman et al., 2003). The *t*-11 C18:1 isomer, which is used for further synthesis of *c*-9,*t*-11 CLA in adipose tissues and mammary gland, is derived from incomplete BH of unsaturated fatty acids (UFA) in the rumen. Linseed oil is one of the richest sources of ALA (Thanh and Suksombat, 2015). Regarding health effects of very long chain n-3 fatty acids (FA) including eicosapentaenoic acid (EPA) and docosahexaenoic acid (DHA), Calder (2014) compiled evidence indicating that these FA could reduce risk of cardiovascular morbidity and mortality, enhance mental development, reduce the burden of psychiatric illnesses in adults and help maintain important roles in the eye and brain structure.

Docosahexaenoic acid, a main FA in marine oil, is responsible for inhibiting ruminal BH of VA into SA, resulting in an increase of *trans* C18:1 available for incorporation in tissue lipids (Lee et al., 2008). Ferreira et al. (2016) concluded that only a small amount of FO inclusion (2.5 g/kg dry matter, DM) in lamb diets was necessary to optimize ruminal concentration of CLA, whereas Lee et al. (2005) found a linear increase in duodenal flow of total CLA with increasing FO up to 40 g/kg DM. Finding a proper amount of FO to replace LO could increase concentrations of *c*-9,*t*-11 CLA, ALA, EPA and DHA in the rumen as well as ruminant meat and milk. Dietary supplementation of FO in combination with LO has been tested in dairy and beef cattle (Brown et al., 2008; Shingfield et al., 2011), but data in goats are scarce. Thus, the aim of this study was to investigate how partial substitution of FO for LO affects feed intake, nutrient digestibility, ruminal fermentation and ruminal FA BH in growing goats fed a diet based on guinea grass.

## MATERIALS AND METHODS

All experimental procedures were conducted following the Ethical Principles and Guidelines for the Use of Animals issued by National Research Council of Thailand. The study was performed at Experimental Farm and Center for Scientific and Technological Equipment, Suranaree University of Technology, Thailand.

### Experiment 1

#### Animals, experimental design and diets.

Four growing male goats (Saanen breed), aged 6 months and weighing 18.13 ± 0.25 kg, were used in this study. Goats were kept in individual wooden cages (1.5 m × 1.0 m × 1.4 m, L × W × H) and had free access to water and a mineral block. The basal diet consisted of concentrate fed in pelleted form and chopped fresh guinea grass offered *ad libitum* (C:F 35:65). Diets were offered in equal amounts twice daily at 07:00 and 17:00 h. Goats were assigned to treatments according to a 4 × 4 Latin square design. The treatment diet consisted of the basal diet supplemented (DM basis) with 25 g/kg DM either LO alone or in combination with tuna FO. Treatments were developed by replacing FO for LO at ratios of 0, 5, 10 and 15 g/kg DM corresponding to FO-0, FO-5, FO-10 and FO-15, respectively. Diets (Table 1) were formulated to meet nutrient requirements of growing male goats (NRC, 2007). Pelleted concentrate was weighed daily into plastic bottles, oil blends added, and then mixed well prior to feeding. Goats were then offered fresh guinea grass for *ab libitum* intake. Oil supplement was daily monitored to confirm that the goats were supplemented with 2.5% DM of added oils in the total ration. Each period lasted for 21 days including 14 days for adjustment and 7 days for sample collection.

**Table 1.** Chemical composition and fatty acid profile of feed, oil, rumen fluid + buffer and treatment

Item^1^	Feed ingredients				Rumen fluid + buffer	Treatment^3^			
	Concentrate^2^	Guinea grass	Linseed oil	Fish oil		FO-0	FO-5	FO-10	FO-15
Chemical composition (% DM unless otherwise noted)									
DM	91.00	21.08				46.91	46.91	46.91	46.91
CP	21.15	11.14				14.28	14.28	14.28	14.28
Lipid	3.52	1.15				4.43	4.43	4.43	4.43
Ash	10.66	10.16				10.08	10.08	10.08	10.08
NDF	35.96	61.47				51.23	51.23	51.23	51.23
ADF	19.11	34.62				28.46	28.46	28.46	28.46
NFC^4^	28.71	16.08				19.99	19.99	19.99	19.99
ME, Mcal/kg DM^5^	3.20	2.09	7.74	7.74		2.61	2.61	2.61	2.61
Fatty acid profile (μg/mg for feeds and oils, μg/mL for rumen fluid + buffer)									
C12:0	6.91	0.12	0.10	0.71	3.09	2.44	2.44	2.44	2.45
C14:0	5.00	0.09	0.60	38.63	9.11	1.78	1.97	2.16	2.35
C16:0	5.17	2.33	55.20	221.75	60.02	4.62	5.45	6.29	7.12
C18:0	1.47	0.35	32.20	63.20	104.27	1.53	1.68	1.84	1.99
*t*-9 C18:1	nd^6^	nd	nd	nd	8.95	–	–	–	–
*c*-9 C18:1	8.28	0.68	178.60	127.31	6.40	7.72	7.47	7.21	6.95
*c*-9,*c*-12 C18:2	7.09	1.83	165.30	16.99	4.12	7.71	6.97	6.23	5.49
C18:3n-3	nd	5.70	557.50	nd	2.67	17.55	14.76	11.97	9.19
C22:0	0.29	0.06	0.73	10.06	nd	0.16	0.20	0.25	0.30
C20:5n-3	nd	nd	nd	82.61	nd	–	0.41	0.83	1.24
C22:6n-3	nd	nd	nd	373.74	nd	–	1.87	3.74	5.61

^1^DM: dry matter; CP: crude protein; NFC: non-fiber carbohydrate; NDF: neutral detergent fiber; ADF: acid detergent fiber, ME: metabolizable energy.

^2^Contained: 32% cassava distillers dried meal, 20% soybean meal, 17.5% corn distillers dried grains with solubles, 10% rice bran, 10% wheat bran, 8% molasses, and 2.5% mineral and vitamin supplement. Mineral and vitamin supplement provided per kg of concentrate including 5,000 IU vitamin A; 2,200 IU vitamin D3; 15 IU vitamin E; 8.5 g Ca; 6 g P; 9.5 g K; 2.4 g Mg; 2.1 g Na; 3.4 g Cl; 3.2 g S; 0.16 mg Co; 100 mg Cu; 1.3 mg I; 64 mg Mn; 64 mg Zn; 64 mg Fe; 0.45 mg Se.

^3^FO-0, FO-5, FO-10 and FO-15: fish oil replaced for linseed oil at ratios of 0, 5, 10 and 15 g/kg, respectively.

^4^Calculated as 100 − (CP + NDF + lipid + ash).

^5^Calculated using values from NRC (2001) tables.

^6^Not detectable.

#### Sampling and measurements.

Dry matter intake (DMI) was determined by weighing daily feed offered and refused during the experiment and correcting for the DM content of each dietary component. Feed samples were pooled and stored at −20°C for further analysis. From d15 to d19, total feces and urine were collected to calculate apparent nutrient digestibility and nitrogen balance. Feces were collected in wire-screen baskets placed under the floor of the cages, and urine was collected through a funnel into plastic buckets containing 50 mL of 10% H_2_SO_4_ to keep the final pH below 3. After recording the weight, 10% proportions of 24 h feces were collected and dried in a forced-air oven at 60°C for 48 h, milled through a 1-mm mesh and stored at −20°C for subsequent chemical analysis. On d21, ruminal fluid samples were collected at 0 and 3 h post morning feeding using a 100-mL syringe. A portion of ruminal fluid was immediately fixed with 10% formalin solution in sterilized 0.9% normal saline (1:9, v:v) for direct counting of protozoa (Galyean, 1989). Another portion was immediately used to determine pH using a digital pH meter (HI-5522, Hanna Instruments, Inc., US). A subsample was also filtered through a clean double layer of cotton cloth, and the liquid fraction was acidified with 1M H_2_SO_4_ (9:1 w/w), centrifuged at 10,000 × *g* for 15 minutes and stored at −20°C for analyses of volatile fatty acids (VFA) and NH_3_-N concentrations.

### Experiment 2

#### Experimental design and treatments.

Assessment of FA BH was carried out *in vitro* using an incubation system with 12 continuous fermenters. The experiment was a completely randomized design including the same four treatments as in Experiment 1. Total added oil alone or in the mixtures was 2.5% DM in each fermenter.

#### Substrates, added oil, and inoculum.

Feeds including guinea grass and the concentrate mix collected from Experiment 1 were used as substrates. Guinea grass and concentrate were mixed at a 65:35 ratio (wt:wt, DM basis). Feed samples were analyzed for FA profiles before conducting the *in vitro* experiment. Oils were prepared and added into *in vitro* fermenters mixed with tween 80 solution (P1754, Sigma-Aldrich, USA). Ruminal contents were obtained before the morning feeding from four male goats, aged 6 months, fed a diet based on fresh guinea grass and 21% crude protein (CP) concentrate (C:F 35:65) twice daily at 07:00 and 17:00 h for a 1-week prior to sampling. Ruminal fluid was transported in four pre-warm thermos flasks to the laboratory within 30 min of collection. Ruminal fluid was filtered through a metal sieve with a pore size of 1-mm to retain small particles under continuous flushing with CO_2_ at 39°C. Fatty acid profiles of mixed substrates, oils and inoculum are presented in Table 1.

#### In vitro incubation.

 Strained ruminal fluid (40 mL) from each goat was added to the fermenter containing warm buffer (160 mL) and mixed substrates (2.0 g). After 30 min, oil solutions were directly added into the fermenters. Cultures were continuously mixed in slow-shaking water bath at 39°C under continuous flushing with CO_2_ gas. Samples for FA analysis (5 mL) were taken at 0, 1, 2, 4, 6, 12 and 24 h. Reactions were immediately stopped by cooling in an ice bath.

### Chemical Analysis

Feed and fecal samples were analyzed for DM, organic matter (OM), CP, ether extract (EE), and ash using standard methods (AOAC, 1998). Crude protein (N×6.25) was determined by the macro-Kjeldahl method (Kjeltec™ 8100, Foss, Denmark), procedure 928.08 of AOAC (1998). Ether extract was determined using petroleum ether in a Soxtec extraction system (Soxtec 8000, Foss, Denmark), procedure 948.15 of AOAC (1998). Neutral detergent fiber (NDF) and acid detergent fiber (ADF) were analyzed following the methods of Van Soest et al. (1991), adapted for the fiber analyzer (Fibertec^TM^ 8000, Foss, Denmark). The concentration of N in acidified urine samples and ruminal NH_3_–N concentration were analyzed by the micro-Kjeldahl method (AOAC, 1998). VFA concentration was determined using a gas chromatograph (Filípek and Dvořák, 2009) equipped with a 30 m × 0.32 mm × 0.15 μm film fused silica capillary column (HP Innowax, AB 002, Agient, USA). Injector and detector temperatures were 250°C. The column temperature was set as follows: 80°C for 5 min followed by increased at 10°C/min to 170°C, then increased at 30°C/min to 250°C and held at 250°C for 5 min. VFA peaks were identified based on their retention times, compared with external standards (acetic acid, propionic acid and butyric acid; Sigma-Aldrich, USA). To analyze FA composition, samples were trans-esterified to methyl esters via a base-catalyzed step followed by an acid-catalyzed step as described by De Weirdt et al. (2013). The FA methyl esters (FAME) were extracted twice with 3 and 2 mL of hexane and pooled extracts were evaporated under N_2_ stream until dryness. The residue was dissolved in 1 mL of hexane and analyzed by gas chromatography (HP 7890A series, Agilent Technology, Palo Alto, CA, USA) equipped with a 100 m × 0.25 mm × 0.2 μm film fused silica capillary column (SP 2560, Supelco Inc, Bellefonte, PA, USA) and a flame ionization detector. The column temperature was kept at 70°C for 4 min, then increased at 13°C/min to 175°C and held for 27 min, then increased at 4°C/min to 215°C and held for 17 min, then increased at 4°C/min to 240°C and held for 10 min. Fatty acids were identified by comparison of retention times with external FAME standards (Supelco 37-Component FAME Mix, Supelco Inc, Bellefonte, PA, USA). The CLA mixture (Sigma–Aldrich, Louis, MO, USA) contained *c*-9,*t*-11 CLA, *t*-10,*c*-12 CLA, *c*-9,*c*-11 CLA, and *t*-9,*t*-11 CLA.

### Statistical Analysis

Data in Experiment 1 were analyzed using the GLM procedure. The statistical model was *Y*_*ijk*_ = *µ*+ *O*_*i*_ + *A*_*j*_ + *P*_*k*_+ *ε*_*ijk*_. Where *Y*_*ijk*_ observation from animal *j*, receiving diet *i* in period *k*; *µ*, the overall of mean; *O*_*i*_, the effect of fish oil level (*i* = 1, 2, 3, 4); A_j_, the effect of animal (*j* = 1, 2, 3, 4); *P*_*k*_, the effect of period (*k* = 1, 2, 3, 4); *ε*_*ijk*_, the residual effect. Mean amounts of FA in the Experiment 2 were statistically analyzed by a PROC MIXED procedure with the statistical model *Y*_*ijk*_= *µ* + *O*_*i*_ + *T*_*j*_ + (*O*×*T*)_*ij*_ + *ε*_*ijk*_, where *Y*_*ijk*_ = the dependent variable; *µ* = the overall mean; *O*_*i*_ = the fixed effect of added oil; *T*_*j*_ = the fixed effect of incubation time; (*O*×*T*)_*ij*_ = the fixed effect of interaction between added oil and incubation time; *ε*_*ijk*_ = the random residual error. Goat inoculum source was considered as a random factor. Orthogonal polynomial contrasts (linear and quadratic) were used to examine treatment effects on response variables. Significant differences among treatment means were statistically compared using Tukey. Statistical tests were performed using SAS University Edition 2019 (SAS Institute Inc., Cary, NC, USA). Significant effect of treatment on least squares means was declared at *P* < 0.05 and tendency was declared at 0.05 ≤ *P* < 0.1.

## RESULTS

### Experiment 1

Linseed oil was rich in ALA (557.50 μg/mg), whereas FO contained (µg/mg) high amounts of FA including EPA (82.61) and DHA (373.74) that were not present in feeds or LO. However, compared with LO, FO also contained high amounts (µg/mg) of some saturated FA consisting of C14:0, C16:0 and C18:0, thus, replacement of FO for LO in the diet also increased their concentrations. Ruminal fluid + buffer contained high amounts (μg/mL) of C16:0 and C18:0.

Intakes of DM, OM, and CP did not differ across diets (Table 2). Replacement of FO for LO in the diet resulted in increased (*P* < 0.01) intakes of C14–C18, EPA and DHA, but linearly decreased (*P* < 0.01) intakes of LA and ALA. Quadratic decreases (*P* < 0.05) in apparent digestibility of DM, OM and CP were observed with increasing levels of FO in the diet. There was a remarkable drop (*P* < 0.01) in OM digestibility when goats were fed FO-15. Increasing dietary FO decreased linearly (*P* < 0.05) nitrogen retention, accounting for −12.90% in FO-15 as compared to FO-0 (Table 3).

**Table 2.** Effect of treatment diets on intakes

Item^1^	Treatment^2^				SEM	*P*-value	Contrast^3^	
	FO-0	FO-5	FO-10	FO-15			*L*	*Q*
Feed and nutrient intake, g/d								
DM	666.37	642.32	650.14	635.33	48.43	0.822	0.615	0.646
OM	596.01	575.32	582.55	567.27	42.88	0.809	0.642	0.631
CP	99.38	97.17	98.25	95.70	4.13	0.652	0.674	0.552
EE	30.05	29.19	29.38	28.44	1.26	0.418	0.579	0.302
EE/DM, %	4.52	4.55	4.53	4.49	0.22	0.986	0.802	0.844
Fatty acid intake, g/d								
C12:0	1.76	1.76	1.77	1.77	0.01	0.709	0.877	0.320
C14:0	1.29^d^	1.41^c^	1.53^b^	1.64^a^	0.02	<0.001	0.002	<0.001
C16:0	3.14^b^	3.59^b^	4.16^a^	4.55^a^	0.20	<0.001	0.083	<0.001
C18:0	1.04^c^	1.12^bc^	1.11^ab^	1.28^a^	0.05	0.003	0.216	0.001
*c*-9 C18:1	5.31^a^	5.02^ab^	4.88^bc^	4.59^c^	0.16	0.004	0.158	0.002
*c*-9,*c*-12 C18:2	5.25^a^	4.63^b^	4.19^c^	3.62^d^	0.15	<0.001	0.008	<0.001
C18:3n-3	11.60^a^	9.34^b^	7.62^c^	5.57^d^	0.48	<0.001	0.004	<0.001
C20:5n-3	0.00^d^	0.27^c^	0.54^b^	0.77^a^	0.04	<0.001	0.001	<0.001
C22:6n-3	0.00^d^	1.20^c^	2.42^b^	3.46^a^	0.18	<0.001	0.001	<0.001
Total FA	30.06	29.19	29.38	28.44	1.26	0.417	0.580	0.302

^1^DM: dry matter; OM: organic matter; CP: crude protein; EE: ether extract; FA: fatty acid.

^2^FO-0, FO-5, FO-10 and FO-15: fish oil replaced for linseed oil at ratios of 0, 5, 10 and 15 g/kg, respectively.

^3^Linear (L) and quadratic (Q) effects of supplemented treatments.

^a-d^Means within a row with different superscripts are significantly different (*P* < 0.05).

**Table 3.** Digestibility and nitrogen balance

Item^1^	Treatment^2^				SEM	*P*-value	Contrast^3^	
	FO-0	FO-5	FO-10	FO-15			*L*	*Q*
Digestibility, %								
DM	71.19^a^	67.59^a^^,^^b^	66.45^a^^,^^b^	63.12^b^	2.13	0.010	0.169	0.005
OM	73.90^a^	69.85^a^^,^^b^	69.22^b^	66.01^b^	1.73	0.004	0.061	0.003
CP	80.52^a^	78.67^a^^,^^b^	77.20^a^^,^^b^	74.91^b^	2.12	0.045	0.521	0.016
Nitrogen balance, g/d								
Intake N	15.90	15.55	15.72	15.31	0.66	0.652	0.674	0.552
Fecal N	3.09	3.30	3.58	3.85	0.43	0.167	0.713	0.052
Urine N	1.80	1.90	1.92	1.87	0.52	0.987	0.766	0.873
Retention N	11.01^a^	10.34^a^^,^^b^	10.22^a^^,^^b^	9.59^b^	0.45	0.023	0.214	0.014

^1^DM: dry matter; OM: organic matter; CP: crude protein; N: nitrogen.

^2^FO-0, FO-5, FO-10 and FO-15: fish oil replaced for linseed oil at ratios of 0, 5, 10 and 15 g/kg, respectively.

^3^Linear (L) and quadratic (Q) effects of supplemented treatments.

^a^,

^b^Means within a row with different superscripts are significantly different (*P* < 0.05).

Replacement of FO for LO in diets had no effect on ruminal pH, NH_3_-N, VFA, and protozoa population at 0 h before feeding (Table 4). However, at 3 h after feeding, total VFA concentration was influenced (*P* < 0.01) by feeding FO, with the lowest value observed in the FO-15 group relative to FO-0. Surprisingly, replacement of FO for LO did not cause any shifts in the proportions of individual VFA (*P* > 0.05). Concerning ruminal microbes, compared with those fed only LO, protozoa population decreased significantly (*P* < 0.01) in goats fed the blended FO and LO diets.

**Table 4.** Ruminal fermentation characteristics

Item	Treatment^1^				SEM	*P*-value	Contrast^2^	
	FO-0	FO-5	FO-10	FO-15			*L*	*Q*
0 h								
pH	6.95	6.82	6.89	6.76	0.21	0.597	0.563	0.575
NH_3_-N, mg/dL	14.12	13.67	13.94	14.14	2.76	0.994	0.810	0.923
Total VFA, mM	83.10	82.71	80.88	82.12	4.32	0.893	0.813	0.540
Acetate, %	73.94	75.11	73.24	73.36	1.23	0.228	0.267	0.094
Propionate, %	17.41	16.08	17.79	17.28	1.09	0.239	0.199	0.198
Butyrate, %	8.64	8.82	8.97	9.35	0.75	0.611	0.934	0.293
Acetate/propionate	4.29	4.70	4.15	4.25	0.37	0.268	0.224	0.164
Protozoa^3^, ×10^6^/mL	1.93	2.00	1.90	1.92	0.13	0.711	0.515	0.409
3 h								
pH	6.66	6.66	6.55	6.66	0.18	0.777	0.822	0.565
NH_3_-N, mg/dL	22.07	21.06	20.90	17.92	2.72	0.261	0.996	0.164
Total VFA, mM	116.09^a^	107.85^ab^	102.84^b^	98.68^b^	4.26	0.006	0.075	0.002
Acetate, %	70.26	71.39	70.29	70.48	0.95	0.373	0.185	0.390
Propionate, %	18.97	18.03	19.05	18.49	0.80	0.332	0.252	0.522
Butyrate, %	10.77	10.58	10.66	11.03	0.31	0.285	0.224	0.312
Acetate/propionate	3.71	3.96	3.70	3.83	0.20	0.318	0.225	0.494
Protozoa, ×10^6^/mL	1.86^a^	1.22^b^	1.15^b^	1.08^b^	0.15	0.001	0.002	0.002

^1^FO-0, FO-5, FO-10 and FO-15: replacement of fish oil for linseed oil at ratios of 0, 5, 10 and 15 g/kg DM, respectively.

^2^Linear (L) and quadratic (Q) effects of supplemented treatments.

^3^Protozoa counts were calculated based on cells per g rumen content.

^a-c^Means within a row with different superscripts are significantly different (*P* < 0.05)

### Experiment 2

**Figure 1.**
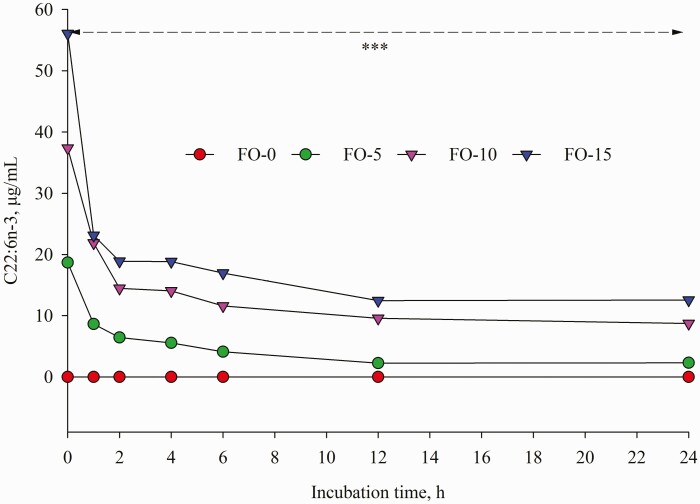
Temporal change of C22:6n-3 concentration during 24 h incubation. Values represent least square means (*n* = 4, SEM = 0.89). ***: *P* < 0.001.

**Figure 2.**
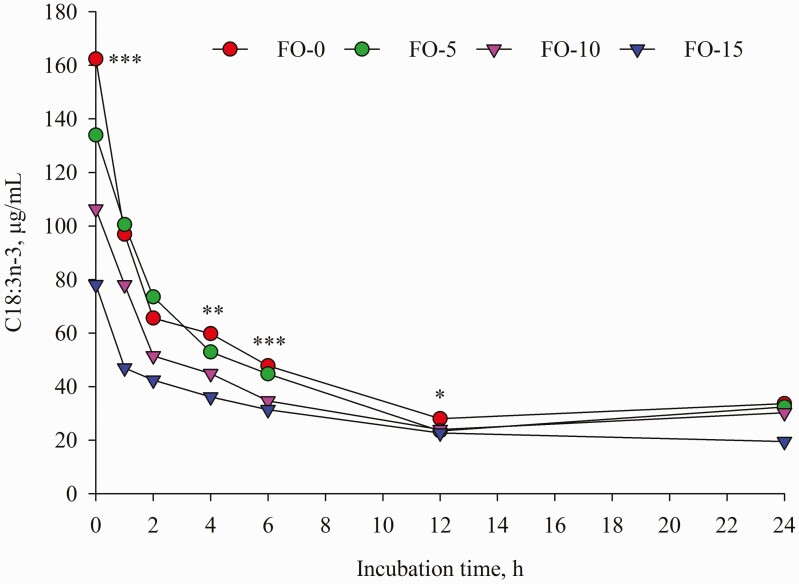
Temporal change of C18:3n-3 concentration during 24 h incubation. Values represent least square means (*n* = 4, SEM = 3.77). *: *P* < 0.05; **: *P* < 0.01; ***: *P* < 0.001.

**Figure 3.**
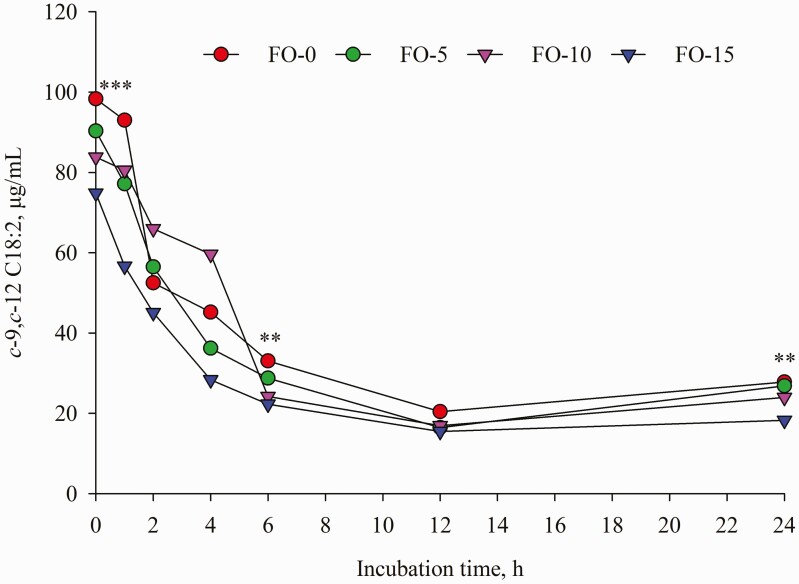
Temporal change of *c*-9,*c*-12 C18:2 concentration during 24 h incubation. Values represent least square means (*n* = 4, SEM = 5.18). **: *P* < 0.01; ***: *P* < 0.001.

**Figure 4.**
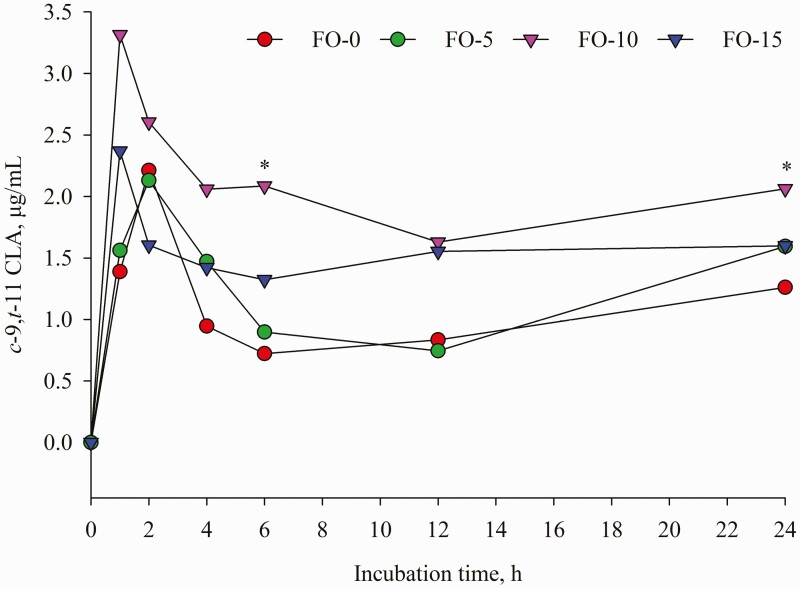
Temporal change of *c*-9,*t*-11 CLA concentration during 24 h incubation. Values represent least square means (*n* = 4, SEM = 0.23). *: *P* < 0.05.

**Figure 5.**
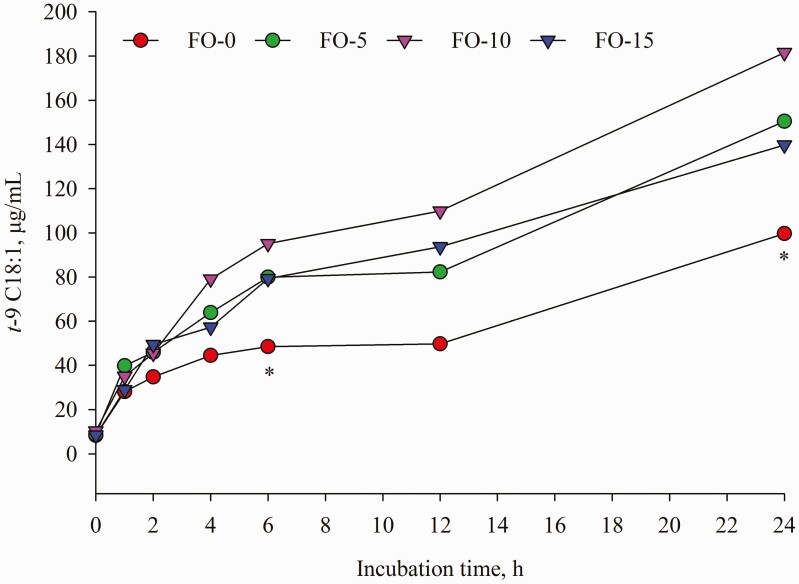
Temporal change of *t*-9 C18:1 concentration during 24 h incubation. Values represent least square means (*n* = 4, SEM = 2.11). *: *P* < 0.05.

**Figure 6.**
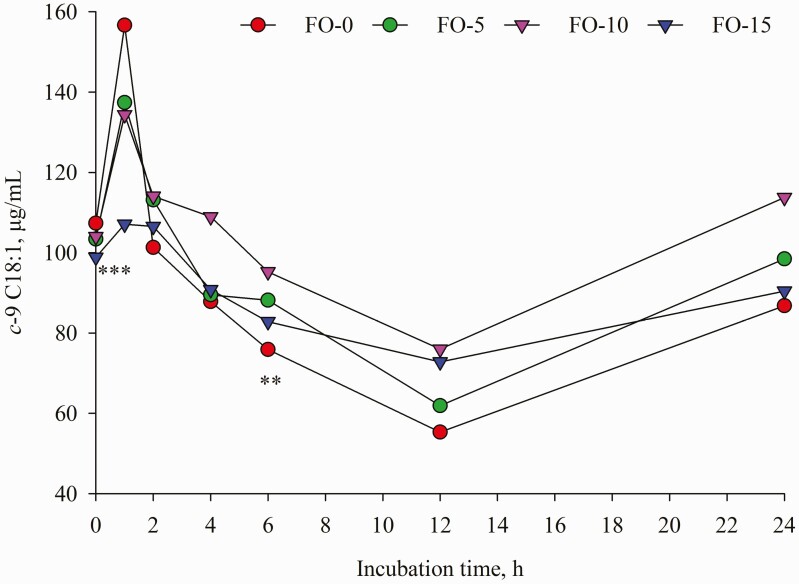
Temporal change of *c*-9 C18:1 concentration during 24 h incubation. Values represent least square means (*n* = 4, SEM = 7.93). **: *P* < 0.01; ***: *P* < 0.001.

**Figure 7.**
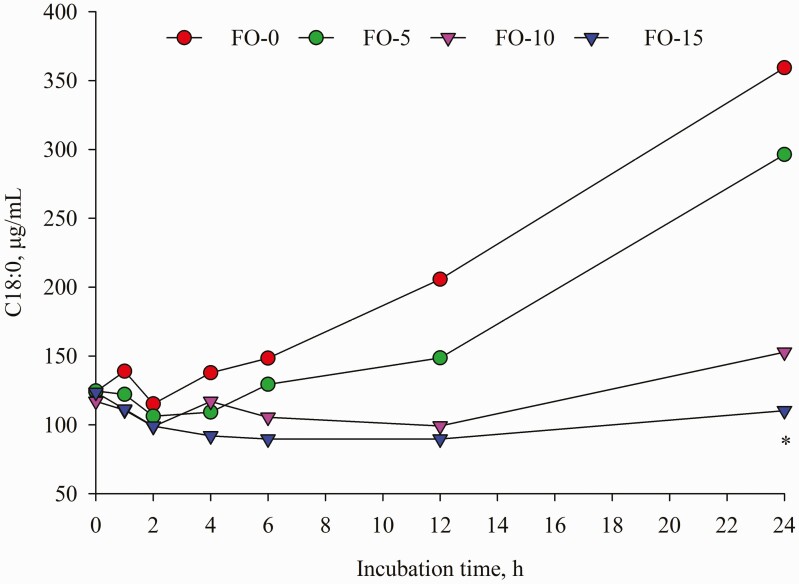
Temporal change of C18:0 concentration during 24 h incubation. Values represent least square means (*n* = 4, SEM = 5.61). *: *P* < 0.05.

Dietary replacement of FO for LO resulted in time-dependent shifts in ruminal FA concentrations characterized by decreases in C18:0, LA and ALA and concomitant increases in *t*-9 C18:1, EPA and DHA (Table 5). Compared with LO inclusion alone, FO and LO blends decreased (*P* < 0.001) mean concentration of C18:0. Further analysis of FA changes during incubation revealed that concentration of C18:0 remained unchanged until 12 h of incubation; however, a different change was detected at 24 h incubation (Figure 7) with the highest amount in FO-0 (175.61 μg/mL) versus the lowest value in FO-15 (102.20 μg/mL). Relative to the amounts at 0 h incubation, C18:0 concentration differed by 189.68, 137.84, 30.49 and −10.75% in FO-0, FO-5, FO-10 and FO-15, respectively. Increasing dose of FO in place of LO linearly decreased (*P* < 0.001) concentrations of ALA and LA (Table 5). Except at 1, 2 and 24 h sampling (Figure 2), the different pattern of ALA concentration remained until end of incubations while the difference (*P* < 0.01) of LA among the treatments was observed only at 0, 6 and 24 h incubation (Figure 3). The FO-10 treatment increased the formation of *c*-9,*t*-11 CLA from the early (1 h) to later stages of incubation. The greatest amounts of *c*-9,*t*-11 CLA in the FO-10 treatment were measured at 6 and 24 h of incubations (*P* < 0.05, Figure 4). Relative to the FO-0 diet, the amounts of *c*-9,*t*-11 CLA with the FO-10 diet increased by 2.39- (at 6 h) and 2.18-fold (at 24 h). Increasing amount of FO in the diets resulted in an increase of *t*-9,*t*-12 C18:2 (quadratic effect; *P* < 0.001), the highest value in FO-10 (5.96 μg/mL) versus the lower value in FO-0 (3.68 μg/mL). Fish oil substitution for LO notably increased (*P* = 0.01) concentration of *t*-9 C18:1 during incubation (Table 5), with the greatest response (*P* < 0.05) detected with FO-10 at 6 h of incubation compared with FO-0 (Figure 5). Concentration of *c*-9 C18:1 was not affected by the replacement of FO for LO, but its concentration was higher (*P* < 0.01) with FO-10 at 6 h incubation relative to FO-0 (Figure 6). Regardless of FO dose, fish oil resulted in linear (*P* < 0.001) increases in concentrations of EPA and DHA (Table 5) and a linear decrease (*P* < 0.001) in DHA concentration over time of incubation (Figure 1).

**Table 5.** The changes in mean amounts (μg/mL) of fatty acid during 24 h incubation

Item	Treatment^1^				SEM	*P*-value^2^			Contrast^3^	
	FO-0	FO-5	FO-10	FO-15		Trt	T	Trt × T	*L*	*Q*
Saturated FA										
C12:0	58.99	56.66	55.77	53.53	11.93	0.552	<0.001	0.037	0.169	0.986
C13:0	3.67	3.08	2.36	2.36	2.10	0.161	<0.001	0.743	0.037	0.516
C14:0	34.96	35.30	35.32	35.45	7.45	0.997	<0.001	0.528	0.849	0.953
C15:0	6.31^b^	6.59^ab^	6.91^ab^	7.08^a^	0.96	0.092^*^	0.002	0.119	0.015	0.803
C16:0	124.27	131.46	133.55	136.70	22.08	0.404	<0.001	0.143	0.112	0.701
C17:0	13.80	13.33	15.31	16.55	4.44	0.105	<0.001	0.986	0.028	0.374
C18:0	175.61^a^	148.03^b^	114.39^c^	102.20^c^	11.03	<0.001	<0.001	<0.001	<0.001	0.344
C20:0	5.13	5.33	5.32	5.25	1.00	0.867	<0.001	0.077	0.693	0.484
Monounsaturated FA										
C14:1	11.05	10.15	9.75	9.68	2.15	0.190	0.159	0.636	0.049	0.391
C15:1	7.73	7.50	7.22	7.28	1.93	0.782	<0.001	0.291	0.364	0.718
C16:1	3.81	3.33	3.61	3.91	0.87	0.195	0.001	0.240	0.510	0.061
*t*-9 C18:1^#^	24.61^b^	31.87^a^	32.72^a^	29.28^ab^	7.34	0.010	<0.001	0.003	0.046	0.004
*c*-9 C18:1	109.22	115.93	108.72	102.09	24.12	0.420	<0.001	0.271	0.276	0.258
Polyunsaturated FA										
*t*-9*,t*-12 C18:2	3.68^c^	5.76^a^	5.96^a^	4.22^b^	1.21	<0.001	<0.001	<0.001	0.240	<0.001
*c*-9,*c*-12 C18:2	70.12^a^	69.42^a^	65.39^a^	52.96^b^	16.70	0.021	<0.001	0.306	0.005	0.135
C18:3n-3	83.09^a^	87.13^a^	65.29^b^	47.54^c^	14.65	<0.001	<0.001	0.001	<0.001	0.002
*c*-9,*t*-11 CLA	1.40	1.70	1.97	1.67	0.83	0.154	<0.001	0.440	0.162	0.088
*t*-10,*c*-12 CLA	0.50	0.54	0.55	0.58	0.45	0.950	<0.001	0.796	0.582	0.955
C20:3n-6	1.26	1.27	1.50	1.32	1.33	0.921	<0.001	0.684	0.754	0.738
C20:5n-3	0.00^d^	2.49^c^	3.82^b^	5.06^a^	0.72	<0.001	<0.001	<0.001	<0.001	0.013
C22:6n-3	0.00^d^	8.87^c^	18.73^b^	24.42^a^	2.66	<0.001	<0.001	<0.001	<0.001	0.026

^1^FO-0, FO-5, FO-10 and FO-15: replacing fish oil for linseed oil at ratios of 0, 5, 10 and 15 g/kg DM, respectively.

^2^Trt: treatment; T: time.

^3^Linear (L) and quadratic (Q) effects of supplemented treatments.

^#^ *t*-11 C18:1 co-eluted with the *t*-9 C18:1 peak.

^a-d^Means within a row with different superscripts are significantly different (*P* < 0.05).

^*^Means within a row with different superscripts are significantly different (*P* < 0.10).

## DISCUSSION

Although Hassanat and Benchaar (2021) reported that feeding increasing doses of LO linearly reduced intake in dairy cows, the lack of effect of treatment diet on DMI in the present study was in agreement with previous studies in lambs and goats (Ferreira et al., 2016; Thanh et al., 2018; Büyükkılıç Beyzi et al., 2020). We speculate that feeding diets that were iso-lipid in the present study contributed to the lack of negative impact on DMI. Ferreira et al. (2016) reported that total digestibility of DM, OM, and CP was not affected in lambs supplemented with fish oil. Relative to previous study, the reduction of nutrient digestibility when diets containing FO were fed in the present study could have been due to the higher amounts of FO used to replace LO. For example, Ferreira et al. (2016) used only 7.5 g/kg FO to substitute for soybean oil in the diet of lambs. Furthermore, supplementation of fish oil rich in EPA and DHA can be harmful to microbial membranes in the rumen and led to reduced number of total bacteria (Huws et al., 2010), which contributed to reduced nutrient digestibility.

A transient reduction in ruminal total VFA concentration after 3 h feeding FO diets indicated that this oil disturbed fermentation by ruminal microbes, which was in agreement with Ferreira et al. (2016). The fact that fish oil inclusion caused a reduction of total VFA concentration without affecting individual VFA proportions implied that concentrations of individual VFA including acetate, propionate and butyrate decreased markedly as fish oil was added into the diet. The lower ruminal VFA production could have been due to lower DM and OM digestibility, likely reflecting on the negative influence of double bonds in EPA and DHA on microbiota. A linear drop in ruminal protozoa population with increasing dose of FO in the diet underscored that FO is highly toxic to ruminal microbes. The greater mitigation of ruminal protozoa population in the animals fed FO and LO blends than those fed only LO seemed a result of synergistic effect of oil combination (Soliva et al., 2004). The observed decrease in ruminal protozoa in this study was a result of oil supplementation rich in long chain UFA. In fact, dietary lipids are almost hydrolyzed in the rumen by microbial lipases, releasing free long-chain FA that may inhibit activity of ruminal microorganisms. Maia et al. (2007) concluded that microbial toxicity of EPA and DHA, main FA in FO, was greater than ALA, predominant FA in LO.

The shift in FA concentrations that occurred during the 24 h incubations (Figure 1–7) indicated that continuous cultures were an adequate model of ruminal activity, and BH of FA was comparable to what occurs in the rumen (Jenkins et al., 2008). Replacement of FO for LO positively influenced ruminal concentrations of ALA, LA, *c*-9,*t*-11 CLA and C18:0 likely due to BH of ALA and LA (Szczechowiak et al., 2016). It is well known that DHA in FO could inhibit the reductase activity of ruminal bacteria responsible for the conversion of VA to C18:0 (Toral et al., 2010), which would allow for the production of *cis* and *trans* C18:1 isomers in the rumen (Laverroux et al., 2011), and use of VA for *c*-9,*t*-11 CLA synthesis by Δ9 desaturase in mammary gland (Shingfield et al., 2013). Although concentrations of 18:1n-9, 18:2n-6 and 18:3n-3 with FO were numerically lower than those in LO, ruminal concentration of *t*-9 C18:1 (Table 5) increased linearly with increasing amounts of FO oil in the diet. Despite FO leading to higher (nearly two-fold) C18:0 content than LO, the lower amounts of ruminal C18:0 with FO-10 and FO-15 demonstrated that replacement of FO for LO at 10 and 15 g/kg DM reduced VA or other unsaturated-C18 hydrogenation to form stearic acid. Similar results were observed in previous studies (Shingfield et al., 2012; Toral et al., 2016; Thanh et al., 2018).

A limitation of the FA protocol in this study was that it could not define the *t*-11 C18:1 peak from other *trans* isomers of similar elution time; therefore, it could not explain the changes of all FA involving the process of LA and ALA BH. The greater amounts of *t*-9 C18:1 with FO diets suggested there was an increase in the conversion efficiency of VA to *t*-9 C18:1 or that the production of VA was large enough to co-elute with the *t*-9 C18:1 peak. Klein and Jenkins (2011) reported that DHA (main FA in FO) can elevate *trans*-18:1 isomers. Kholif et al. (2020) reported that inclusion of EPA-rich microalgae in the diet of dairy goats increased *t*-9 C18:1 concentration in milk fat. The higher amount of *c*-9,*t*-11 CLA in FO-10 at 6 h after incubation suggested that use of FO at this level in place of LO not only inhibited the conversion of VA to C18:0, but also prevented hydrogenation of *c*-9,*t*-11 CLA to VA. However, feeding FO-15 elicited a modest improvement in *c*-9,*t*-11 CLA compared with FO-10 largely because LO-15 had a lower amount of LA. Feeding fish oil in dairy goats increased milk *c*-9,*t*-11 CLA (Büyükkılıç Beyzi et al., 2020). A reduced amount of ruminal EPA and DHA after 24 h incubation reflected extensive BH of these FA in the rumen (Shingfield et al., 2012; Kairenius et al., 2015). A linear increase in amount of ruminal EPA and DHA over time of incubation with increasing FO level in the diet indicated that BH of EPA and DHA was partially inhibited when levels of these FA was high. Ferlay and Chilliard (2020) reported an increased milk DHA content in dairy cows fed 2.5% fish oil. This was also in agreement with the findings of AbuGhazaleh and Jenkins (2004), who noted a decrease in the percentage disappearance of EPA and DHA in batch cultures with increased FO supplementation.

## CONCLUSION

Substitution of FO for LO decreased nutrient digestibility and ruminal total VFA concentration without affecting individual VFA proportions. Replacement of FO for LO from 5 to 15 g/kg DM remarkably decreased ruminal protozoa populations. Increasing dose of FO in the diet resulted in decreased mean amounts of C18:0, LA and ALA but increased mean amounts of EPA and DHA. Mean amount of *c*-9,*t*-11 CLA was not change by feeding FO, but the higher concentration of this FA was detected in FO-10 at 6 and 24 h incubation. To improve ruminal concentrations of EPA, DHA, *c*-9,*t*-11 CLA and reduce C18:0 concentration without or less affecting digestibility and ruminal fermentation, a dietary supplementation of 15 g/kg LO and 10 g/kg FO would be suitable.
